# IL-33 and IL-18 in Inflammatory Bowel Disease Etiology and Microbial Interactions

**DOI:** 10.3389/fimmu.2019.01091

**Published:** 2019-05-14

**Authors:** Michelle A. Williams, Amy O'Callaghan, Sinéad C. Corr

**Affiliations:** Department of Microbiology, School of Genetics and Microbiology, Moyne Institute of Preventative Medicine, Trinity College Dublin, Dublin, Ireland

**Keywords:** IBD, IL-18, IL-33, colitis, microbiota

## Abstract

The IL-1 cytokines are a newly expanded family, with each of its 11 members playing an important role in health and disease. Typically acting as pro- or anti-inflammatory mediators of first-line innate immunity, their production is particularly important in the context of mucosal defenses, through handling breach of the delicate epithelial barrier and mediating a local immune response to invading pathogens. Mucosal immunity is often aberrantly orchestrated in intestinal diseases, such as Inflammatory Bowel Disease (IBD). Various studies have pointed to IL-1 cytokines as being important players in IBD with context-dependent roles, either through promoting auto-inflammatory mechanisms, or alleviating disease through protection against breach of pathogens across the epithelial barrier. This mini-review will succinctly examine the role of IL-1 family members in IBD, with a special focus on the recently described IL-33 as well as IL-18, and will explore the disease models within which these cytokines have been studied. Furthermore, we will examine the evidence of interplay of these cytokines with the gut microbiota, with hopes of summarizing our current knowledge of these family members and their potential for unraveling novel molecular mechanisms of IBD pathology.

## Introduction

The IL-1 family of cytokines primarily act on innate immunity to initiate inflammation in the face of local insult, thus playing a particularly important role in the pathophysiology of mucosal diseases. Members of the IL-1 family of cytokines play important, yet context-dependent roles in intestinal homeostasis and inflammation. In this review we will examine two IL-1 family cytokines, IL-33 and IL-18, in the context of mucosal immunity and with a particular focus on the pathogenesis of IBD, a chronic inflammation of the intestinal mucosa. This review will explore their role in host immunity, and describe associations of these cytokines with the host microbiota, a major component in IBD etiology. Together this review will summarize our knowledge of these newly described cytokines, and present an outlook on their individual and complex roles in IBD (Table 1).

**Table 1:** Known effects of IL-18 and IL-33 with implications for IBD pathogenesis.

**Cytokine**	**Experiment**	**Observed effect**	**Implication for cytokine in IBD**
**IL-18**			
	Activity blocked by IL-18 binding protein	Reduced small intestinal pathology caused by *T. gondii* infection *ex vivo* (25)	Detrimental
	Activity blocked by IL-18 binding protein	Attenuated DSS-colitis (26)	Detrimental
	Increased expression	Decreased butyrate producers in microbiota, with subsequent exacerbation of colitis (27)	Detrimental
	Knock-out	Protected against DNBS-induced disease in both single KO and double KO with IL-1β (28)	Detrimental
	Overexpression in enterocytes	GI tract overexpression promoted eosinophilic inflammation in rats (29)	Detrimental
	Targeted inhibition	Inflammatory mucositis alleviated in mice (30)	Detrimental
	Receptor knock-down	Protected against DSS-induced colitis in mice (31)	Detrimental
	Treatment with recombinant IL-18	Increased neutrophil transmigration across Caco_2_ monolayer through Occludin loss (32)	Detrimental
**IL-33**			
	Deletion of nuclear sequestration signal	Lethal inflammation dependent on signaling through ST2 (33)	Detrimental
	Knock-out	Impaired recovery from extended DSS-colitis in mice (34)	Protective
	Receptor knock-out	Reduction in myeloid precursors of inflammation (35)	Detrimental
	Receptor signaling blockade	Alleviation of colitis in SAMP mice (23)	Detrimental
	Treatment with recombinant IL-33	Alleviation of TNBS colitis in mice through polarization of homeostatic M2 macrophages (19)	Protective
	Treatment with recombinant IL-33	Alleviation of chronic colitis in mice, reduced bacterial translocation (36)	Protective
	Treatment with recombinant IL-33	Reduced colitis severity in mice in an IL-10 dependent manner (37)	Protective
	Treatment with recombinant IL-33	Aggravated acute colitis (24)	Detrimental

## IL-33

### IL-33: An Alarmin in Mucosal Immunity

The IL-1 family member IL-33 plays a unique and essential role in mucosal, front-line immunity. Previously known as IL-1F11, IL-33 is a relatively newly described cytokine, with origins tracing back to 2005 (1). It was discovered after the characterization of its cognate receptor, suppressor of tumorigenicity 2 (ST2) (2). IL-33/ST2 signaling not only acts as a front-line herald of tissue damage, but also links innate and adaptive immunity at the host mucosae through potent induction of a type 2 response in T cells, innate lymphoid cells (ILCs) and macrophages (3–5). Despite potentially playing an important role as a mediator of mucosal immunity, and being suggested as a drug target for various disorders, there are currently no IL-33-based therapies for intestinal disease. This presents an interesting opportunity for study of this cytokine and its role in IBD.

The most well-characterized aspect of IL-33 biology is its role as an alarmin: a molecular “fire-alarm” at the barrier tissues of the body, driving inflammatory and fibrotic processes during acute mucosal breach due to cell injury (6). IL-33 is constitutively expressed in epithelial and endothelial cells, and following translation is stored as a full-length, biologically active molecule in the nucleus where it binds to chromatin (7). Following lysis of the cell through destructive mechanisms, IL-33 in the nucleus is immediately available to act as an early signifier of damage, through recruitment of neutrophils, eosinophils, natural killer (NK) cells, and by amplifying a type 2 (Th2, ILC2, M2-like macrophage) response in order to initiate fibrosis and wound healing (8, 9). Interestingly, not only being important for primed release of the cytokine, sequestration of IL-33 in the nucleus allows it to act as a transcriptional regulator, where it can bind to the p65 subunit of NFκB to activate endothelial cells (10). Unlike other members of the IL-1 family, IL-33 does not require processing through an inflammasome in order to achieve biological activity and in fact is inactivated by caspase cleavage (11). However, N-terminal cleavage by neutrophil elastase and cathepsin G proteases, which are found in the microenvironment during inflammation, can increase its potency (12). This again highlights the primary role of IL-33 in orchestrating the response to cellular destruction.

### IL-33 in Intestinal Disease

Expression of IL-33 and its receptor ST2 has been well-established in the GI tract, being an important amplifier of innate immunity at the gut mucosa (13). While IL-33 is expressed largely at the mucosae and in myofibroblasts, its receptor is expressed mainly on immune cells, such as ILC2s, Tregs, T helper cells, and CD8+ T cells (14) This allows IL-33/ST2 signaling to act as a bridge between tissue damage and immune system orchestration, which may be a critical component in intestinal immunity. In an experiment whereby the N-terminus of IL-33 was altered such that it could not associate with chromatin, the result was the formation of a whole-body inflammatory response with splenomegaly, increased lymph node infiltration and indeed development of colitis (7). This response was then ablated by knock-down of the ST2 receptor. Not only does this demonstrate the importance of nuclear sequestration of IL-33 but also highlights the potency of IL-33/ST2 signaling in the acute inflammatory response and a potential role in intestinal disease.

IL-33 presents an interesting role in IBD, which is perhaps complicated by the divergent immune pathophysiologies of Crohn's disease (CD) and Ulcerative Colitis (UC). Studies of patient biopsies have shown an increase in IL-33 levels in patients with active IBD, in particular UC, compared with healthy controls (15). Interestingly, UC-associated IL-33 is found in myofibroblasts, which tend to localize at the base of inflamed ulcerations during disease (16). Furthermore, blockade of IL-33/ST2 signaling has been shown to alleviate active disease, suggesting a pathogenic role for the cytokine (17).

In contrast with these findings, IL-33-deficient mice have been shown to be highly susceptible to colitis and colorectal cancer, which would suggest a role as an important protective mediator of intestinal immunity (18). This offers a contradictory and complex role for IL-33 in IBD. This is evidenced by results from various mouse models of disease, including but not limited to the most widely used methods of dextran-sodium sulfate (DSS)-induced and trinitrobenzene sodium (TNBS)-induced colitis. While DSS-induced colitis is largely T cell independent and mediated by chemical damage, TNBS colitis development is dependent on induction of a Th1 response. Given its association with boosting type 2 immunity, IL-33 may act as a “balancing” cytokine in TNBS colitis. This has been shown in a model of TNBS colitis, whereby recombinant IL-33 administration was shown to attenuate disease development through induction of M2-like macrophage polarization (19). Furthermore, IL-33 has been found to ameliorate TNBS colitis, in a manner that was Foxp3 dependent, through promoting a Th2 and Treg response (20). Agreeing with this, IL-33 has been shown to enhance Foxp3+ Treg cell expansion in the intestine via TGF-β (21). Attempting to clarify this, another study employed the use of SAMP colitis mice, which are characterized by development of T cell-driven enteric inflammation. SAMP mice show an early Th1 stage, which is followed by establishment of a chronic Th2-mediated disease (22). UC-like disease in SAMP mice was found to correlate with expression of full-length IL-33 in IECs, and blockade of the IL-33/ST2 signaling pathway was beneficial (23). This demonstrates a somewhat complex role for IL-33 in IBD, wherein its effector role may be determined by the T cell response pattern and intrinsic differences in CD and UC immunology.

In the context of DSS-induced colitis, IL-33 plays a varying role depending on the temporal stage of the disease. For instance, one particular study showed that administration of recombinant IL-33 exacerbated acute colitis, but ameliorated colitis in a chronic model of disease in a manner dependent on amphiregulin-EGFR signaling (24). IL-33 induced neutrophil influx during both stages of disease, which may have contributed to its exacerbating effects on acute colitis through nitric oxide (NO) immunopathology, but was shown by the authors of the study to reduce translocation of pathobionts across the impaired epithelium during chronic disease. This divergence, even through use of the same disease agent, points toward IL-33 expression patterns as an important tool for study of the early and late immune response in IBD, suggesting that despite its role in promoting acute inflammation, it may act to limit chronic inflammation in long-term disease.

## IL-18

### IL-18 as a Pro-inflammatory Regulator

The role of IL-18 in intestinal disease is largely related to its activity in regulating pro-inflammatory responses. Discovered in 1989 in mouse serum following challenge with bacterial LPS, IL-18 was first identified as a booster of IFNγ activity produced by monocytic cells (38). To compare it with its sister cytokines, IL-18 most similarly resembles IL-1β, in both structure and their association with the inflammasome, a proteolytic complex through which IL-1β and IL-18 precursor molecules become biologically active by caspase-1 cleavage. GI commensals are critically important in regulating intestinal inflammasome assembly, and this has been demonstrated in new-born mammals that feature progressive microbial colonization, accompanied by intestinal barrier formation and immune system maturation. This perhaps further highlights a potential role for aberrant IL-18 in IBD, a disease characterized by an excessive inflammatory response to microbial products. Furthermore, loss-of-function single nucleotide polymorphisms (SNPs) in the IL-18 gene result in an imbalance of the Th1/Th2 response, which promotes host susceptibility to CD (39). Other meta-analysis studies have supported this observation (40).

IL-18 is generally a pro-inflammatory mediator, and its production may be a key etiological factor for patients with IBD (41, 42). Pro-IL-18 is produced by a wide range of cell types, including epithelial cells, myeloid cells and lymphocytes, and following inflammasome activation carries out a wide range of effector functions, including promoting the production of IFNγ, priming of NK cell cytotoxicity (43) and stimulating the differentiation of Th1 cells (44). Despite this, the NLRP6 inflammasome itself is a known regulator of colonic homeostasis, predominantly expressed in intestinal epithelial cells (IECs) with a key role in mucosal renewal, proliferation and secretion (45). Interestingly, deficiency in the NLRP6 inflammasome is detrimental in DSS-induced colitis, in a manner related to insufficient IL-18. Likewise, caspase-1 deficiency is linked to increased DSS severity. These reports would suggest that while IL-18 is a pro-inflammatory mediator, its baseline activity is important for intestinal integrity through unknown mechanisms.

On the other hand, IL-18 has been also shown to contribute to the breakdown of the mucosal barrier, provoking inflammation and amplifying damage elicited to the intestinal epithelium during disease. Clinical studies have correlated increased epithelial secretion of IL-18 with increased severity of IBD (46). This study was supported by Nowarski et al., where deletion of IL-18 receptor (IL-18R) from IECs shielded mice from DSS-induced colitis (31). Transgenic mice deficient in IL-1β, IL-18 or both cytokines protected against TNBS colitis induction in mice (28). Thus, the double knockout increased the protective effects against intestinal inflammation, perhaps due to the inhibition of two converging inflammatory pathways. IL-18 production is elevated in IECs following infection with human immunodeficiency virus (HIV), causing IEC apoptosis through the activation of caspase-1 and caspase-3 (47), two programmed cell death proteases. This study also described how IL-18 disrupts the tight junctions that maintain intestinal epithelium integrity. Collectively, these results suggest that IL-18 overproduction generally contributes to an increase in the permeability of the intestinal monolayer, exacerbating intestinal inflammation. Contribution to inflammation in such a regard presents an opportunity to alleviate IBD symptoms, which is being exploited through development of a monoclonal antibody against IL-18. This is currently in phase one of clinical trials (clinicaltrials.gov ID: NCT01035645).

## IL-18 and IL-33 in Host-Microbe Interactions

The current working hypothesis for the etiology of IBD focuses on the loss of host tolerance for the resident microbiota. As such, an exploration of the role of IL-18 and IL-33 should critically examine their known associations with the microbiota, and the ways in which this may alter host pathology during IBD. The role of IL-18 in this regard is well-established in the literature. Indeed, intestinal IL-18 levels progressively increase over the first 5 weeks of post-natal development, harmonizing with microbial colonization (48). Levy et al., demonstrate how gut microbiota bi-products, such as metabolites, influence NLRP6 inflammasome signaling, IL-18 epithelial cell secretion and downstream secretion of anti-microbial peptides (AMPs) (49). This study is supported by Elinav et al., where NLRP6-inflammasome deficient mice had impaired production of IL-18 and concomitant dysbiosis, characterized by an expansion of the bacterial phyla Bacteroidetes (*Prevotellaceae*) and TM7 (50). This deficiency in NLRP6 and IL-18 results in a failure to produce AMPs, which are essential in mediating pathogenic bacterial clearance. Recently, NLRP6 was shown to prevent the colonization of IBD-inducing bacteria, the mucolytic *A. muciniphila*, which can induce colitis in both specific pathogen free and germ free (GF) *IL10*^−/−^ mice. This is mediated by IL-18 (51).

Interestingly, GF mice colonized with a complex human gut-derived microbiota see a transient increase in IL-18 without tissue damage or inflammation, suggesting an ongoing homeostatic response (52). IL-22 is a classic Th1-cell-associated pro-inflammatory cytokine that exacerbates ileitis following infection with protozoa, e.g., *Toxoplasma gondii* (53). Interestingly, IL-22 knockout mice were associated with reduced production of Th1-promoting IL-18. IL-22 not only influenced the expression of IL-18 messenger RNA (mRNA) in IECs after infection with *Toxoplasma gondii* or *Citrobacter rodentium* infection, but was found to promote a homeostatic production of active IL-18 in the ileum (54), which not only is crucial for host defense but also contributes to inflammation, while assisting in microbial clearance. This suggests a double-edged sword role for IL-18, whereby it contributes to inflammation but in doing so, protects the host from pathogenic invasion.

Conversely, very little is currently known about IL-33 crosstalk with the gut microbiota, however some studies have suggested a potential for interaction. One such study in SAMP mice showed that IL-33 is induced in ex-GF mice following transplantation of commensals (55). Furthermore, IL-33 expression has a number of known interactions with the microbial-sensing Toll-like receptors (TLRs), and is indeed induced by pathogen-associated molecular patterns (PAMPs), perhaps as a protective mechanism of the host (56). Further highlighting its potential for enabling host-microbiota crosstalk, IL-33/ST2 interactions can repress the TLR2 pathway through sequestration of the adaptor molecule MYD88 (57).

The susceptibility of IL-33 deficient mice to colitis has been shown to be a result of the ability of IL-33 to promote IgA production from B cells, which notably plays a major role in maintaining microbial homeostasis in the intestine (18). This suggests that IL-33 may indirectly alter the microbiota to protect against colitis through promotion of IgA production, which is already known to be a protective factor in IBD. Supporting this, IL-33 deficient mice developed dysbiosis, characterized by increased levels of mucolytic and colitogenic bacteria, which drastically altered the microbial landscape of the gut making the mice more susceptible to colitis. This effect was found to be dependent on IL-1α release, and colitis susceptibility in IL-33 deficiency could be reversed through either reconstitution of a homeostatic microbiota or by IL-1α ablation. This provides a potential role for IL-33 to quell the colitogenic effects of its more sinister sibling cytokines.

## Closing Remarks

In summation, the IL-1 family of cytokines are regulators of mucosal immunity in a manner that appears highly context-dependent. IL-18 itself is a pro-inflammatory mediator capable of exacerbating disease, despite evidence that it can promote homeostasis in some circumstances. The role of IL-33 in IBD is more complex, and perhaps related to disease stage. While both of these cytokines promote early pro-inflammatory responses to effect front-line protection against mucosal breach and pathogenic invasion, their baseline expression is indeed important for the maintenance of overall intestinal integrity. As a result, poorly timed or excessive production of bioactive IL-1 family members may provide a key step in IBD development, with vast potential for therapeutic intervention.

## Author Contributions

MW, AO, and SC developed, wrote, and revised the content in this manuscript.

### Conflict of Interest Statement

The authors declare that the research was conducted in the absence of any commercial or financial relationships that could be construed as a potential conflict of interest.
